# Impact of bundle implementation on the incidence of peri/intraventricular hemorrhage among preterm infants: a pre-post interventional study

**DOI:** 10.1590/1516-3180.2020.0412.R1.28012021

**Published:** 2021-05-10

**Authors:** Cristiane Akemi Koyama Wallau, Daniela Testoni Costa-Nobre, Ana Teresa Figueiredo Stochero Leslie, Ruth Guinsburg

**Affiliations:** I MSc. Postgraduate Student. Neonatal Division, Department of Pediatrics, Escola Paulista de Medicina, Universidade Federal de São Paulo (EPM-UNIFESP), São Paulo (SP), Brazil.; II MD, PhD. Adjunct Professor, Neonatal Division, Department of Pediatrics, Escola Paulista de Medicina, Universidade Federal de São Paulo (EPM-UNIFESP), São Paulo (SP), Brazil; III MD, MSc, PhD. Adjunct Professor, Neonatal Division, Department of Pediatrics, Escola Paulista de Medicina, Universidade Federal de São Paulo (EPM-UNIFESP), São Paulo (SP), Brazil.; IV MD, PhD. Full Professor, Neonatal Division, Department of Pediatrics, Escola Paulista de Medicina, Universidade Federal de São Paulo (EPM-UNIFESP), São Paulo (SP), Brazil

**Keywords:** Quality of health care, Intracranial hemorrhages, Infant, premature, Infant, newborn, Intraventricular hemorrhage, Peri-intraventricular hemorrhage, Bundle

## Abstract

**BACKGROUND:**

Peri/intraventricular hemorrhage (PIVH) is a frequent cause of death and morbidity among preterm infants. Few studies have addressed the use of bundles for preventing PIVH.

**OBJECTIVE:**

To evaluate the efficacy of a bundle of interventions designed to decrease the incidence of intraventricular hemorrhage at hospital discharge among preterm infants.

**DESIGN AND SETTING:**

Pre-post interventional study with retrospective and prospective data collection performed before and after bundle implementation in the neonatal intensive care unit of a university hospital.

**METHODS:**

Infants with gestational age < 32 weeks without malformations, who survived > 6 days were included. The bundle consisted of the following actions during the first 72 hours of life: maintenance of head in neutral position with the body in supine position, minimal handling, including delay of lumbar puncture until after 72 hours and absence of respiratory therapy maneuvers. Cranial ultrasound was performed on days 3, 7 and 28, or later if needed. The effect of the bundle was analyzed through logistic regression and results were adjusted for confounding variables.

**RESULTS:**

167 infants met the inclusion criteria; 146 (87%) were analyzed. Bundle implementation was associated with decreased chances of PIVH at hospital discharge (odds ratio 0.29; 95% confidence interval 0.13-0.65). Cerebrospinal fluid collection within the first 72 hours increased the odds of PIVH of any grade during the hospital stay fourfold, after adjustment for all variables included in the model.

**CONCLUSION:**

Implementation of a bundle of interventions to avoid intraventricular hemorrhage was effective for decreasing the incidence of all grades of PIVH in preterm infants.

## INTRODUCTION

The survival of preterm infants reflects the quality of prenatal and labor and delivery care, as well as the infrastructure available for neonatal care. According to a report published by the World Health Organization in 2012, titled “Born Too Soon: The Global Action Report on Preterm Birth”,^1^ Brazil ranks 10^th^ highest in the number of premature live births and 16^th^ highest in the number of deaths associated with complications of prematurity. Adequate and effective care in the neonatal intensive care unit (NICU) might modify the short, medium and long-term prognoses^2^ for infants who require long hospital stays, especially for those who are extremely preterm.

Peri/intraventricular hemorrhage (PIVH) is a frequent cause of death and morbidity among preterm infants and contributes to an adverse neurological prognosis.^3^^,^^4^^,^^5^ Several studies have sought to identify risk factors associated with PIVH in general, and in grade III and IV lesions in particular,^6^ in order to establish preventive strategies for this condition.^7^ However, because of the complex and multifactorial etiopathogenesis of PIVH, adoption of preventive measures alone is not expected to have much impact on the incidence of this severe neonatal complication.^8^

The outcomes from interventions aimed at improving the quality of hospital care indicate that the use of bundles, i.e. sets of simultaneously applied measures, reduces the incidence of infections,^9^^,^^10^^,^^11^ and central catheter-related late neonatal sepsis in particular, along with reducing other conditions particular to preterm infants.^12^^,^^13^ However, few studies have addressed the use of bundles for prevention of PIVH.^8^^,^^14^^,^^15^ Carteaux et al.^14^ implemented a set of practices to reduce the incidence of intracranial hemorrhage and periventricular leukomalacia among very low birth weight infants, although the impact of those measures was not reported. Schmid et al.^8^ implemented a bundle of measures targeting delivery care and the initial care of neonates in the delivery room and neonatal intensive care unit (NICU) that emphasized minimal handling, and they reported that these measures reduced the incidence of PIVH by 50% among preterm infants with birth weight under 1,500 g. A nursing intervention bundle applied in two Dutch centers reduced the risk of severe PIVH, and it was also associated with a lower risk of any degree of IVH, cystic periventricular leukomalacia and/or mortality.^15^

## OBJECTIVE

Thus, the aim of the present study was to establish whether a bundle of clinical measures implemented during the first 72 hours of life among preterm infants with gestational ages less than 32 weeks could reduce the incidence of PIVH of any grade during the hospital stay.

## METHODS

This was a pre-post interventional study with retrospective (pre) and prospective (post) data collection performed before and after implementation of a bundle of measures at the NICU of a university hospital. The study was approved by the institutional research ethics committee (protocol number: 226.656; approved on March 22, 2013). The study periods were as follows:

1. Data were retrospectively collected before bundle implementation, over a period covering from March 2009 to April 2011. Thus, informed consent for this period was waived.
2. Data were prospectively collected after bundle implementation, from May 2011 to April 2013. For these data, informed consent was required.

All newborn infants with gestational ages less than 32 weeks, as established according to the best obstetrical estimate, without lethal congenital abnormalities or malformations of the central nervous system, were included. Infants who died and/or who underwent surgical procedures within the first 168 hours of life were excluded.

The presence of PIVH was investigated using head ultrasound (US) on days 3, 7 and 28 of life, and later as indicated by the medical staff. The scans were performed using the Acusson X300 US device (Siemens, Erlangen, Germany), with a multifrequency micro-convex 8-5 MHz transducer. The presence and severity of PIVH was assessed in accordance with the methods of Papile.^6^ All US examinations were performed by specialized pediatric radiology staff, and the reports were reviewed by the supervising radiologist.

The frequency of PIVH of any grade and the frequency of grade III/IV lesions among newborn infants with gestational ages less than 32 weeks and without malformations, who survived for more than 12 hours, were 64% and 45%, respectively in the NICU of the present study in 2010. Assuming that the bundle of measures would reduce the incidence rates of PIVH of any grade and grade III/IV lesions to the average seen among 20 hospitals included in the Brazilian Network of Neonatal Research^16^ (35% and 18%, respectively), with power of 90% and two-tailed alpha error of 5%, the required sample size for the periods before and after bundle implementation was 60 individuals.

The bundle implemented within the first 72 hours of life among all the eligible infants consisted of the following procedures: 1) maintaining the infant in dorsal decubitus and neutral elevated head position;^14^^,^^15^^,^^17^ 2) avoidance of physical therapy maneuvers and aspiration of the tracheal cannula;^18^^,^^19^ 3) postponing cerebrospinal fluid (CSF) collection for sepsis work-up until after 72 hours of life;^14^^,^^20^ 5) assessment of daily weight only after the first 72 hours of life; and 6) reinforcement of minimal handling and environmental policies.^14^^,^^15^

Demographic data on the mothers and newborn infants were collected. Advanced resuscitation was defined as the need for tracheal intubation with concomitant chest compression and/or medication. Clinical severity was evaluated using the Score for Neonatal Acute Physiology, Perinatal Extension, Version II.^21^ Neonatal morbidity was defined as the occurrence of at least one of the following events in the first seven days of life: hypothermia (body temperature < 36 °C); metabolic or respiratory acidosis (arterial blood pH < 7.20); hypocapnia or hypercapnia (pCO_2_ < 40 and > 60 mmHg, respectively, in an arterial blood sample); respiratory distress syndrome; air leak syndrome; apnea (pauses > 20 seconds or shorter pauses accompanied by bradycardia and/or cyanosis); hypotension (need for volume expansion and/or use of vasoactive drugs); patent ductus arteriosus (PDA) on echocardiogram requiring pharmacological and/or surgical treatment; hypoglycemia (capillary glycemia < 40 mg/dl); thrombocytopenia (< 150,000/mm); hemorrhagic disorders (active bleeding requiring intervention); and pain (score on validated scales compatible with the presence of pain).

Data collection and monitoring were performed every two days. Adherence to the bundle was defined as keeping the infants in dorsal decubitus with neutral head position and not performing physical therapy maneuvers, cannula aspiration or CSF sample collection within the first 72 hours of life, as documented in the infants’ medical records by the medical, nursing and physical therapy staff.

Neonatal characteristics and variables associated with adherence to the bundle, stratified in relation to the presence of PIVH, were analyzed by means of logistic regression for the outcome of PIVH of any grade during the hospital stay. As a sensitivity analysis, PIVH of any grade at three days of age was also analyzed. Two models were fitted for each outcome, in which all variables that had a P-value < 0.20 on univariate analysis were included as independent variables. In addition, model one also included a “period” variable indicating whether the infants received the bundle. In model two, the “period” variable was removed, and the following bundle-related variables were included: head position (adequate or inadequate); body position (adequate or inadequate); tracheal aspiration (present or absent); respiratory physical therapy maneuvers (present or absent); and CSF sample collection before 72 hours of age (present or absent). Non-significant variables were removed from the model one at a time, and the model goodness-of-fit was tested using the Hosmer-Lemeshow test in SPSS 21.0 0 (IBM SPSS Statistics for Windows, version 21.0; IBM Corporation, Armonk, NY, United States).

## RESULTS

**Figure 1.**
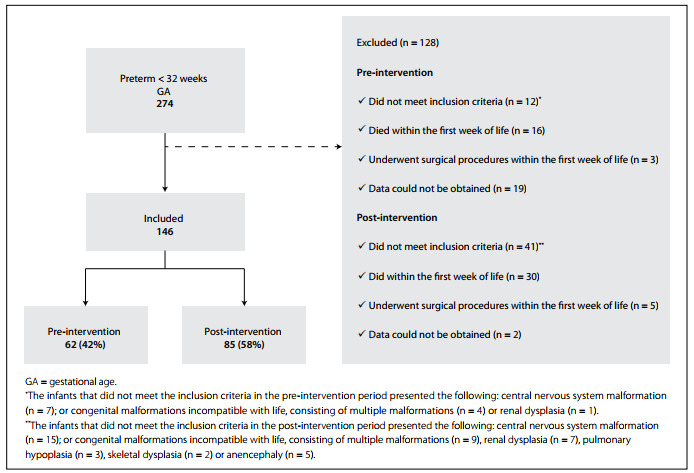
Study population flow chart.

A total of 274 infants with gestational ages less than 32 weeks were born during the study period, and 221 (81%) met the inclusion criteria (Figure 1). Of these, 46 (21%) were excluded because they died within the first week of life, and eight (4.5%) were excluded because they underwent surgical procedures. Among the remaining 167 eligible infants, data could not be obtained for 21 (12.6%). Consequently, 146 infants were included in the analysis: 61 (42%) during the pre-intervention period and 85 (58%) during the post-intervention period.

The mothers’ and newborn infants’ characteristics stratified according to intervention group are described in Table 1; no significant differences were found between the groups.

**Table 1.** Characteristics of 146 newborn infants and their mothers grouped according to study period: before or after implementation of a bundle of measures to prevent peri/intraventricular hemorrhage

	Pre-bundle (n = 61)	Post-bundle (n = 85)	P-value
Maternal characteristics			
Hypertension	23 (37.7%)	28 (32.9%)	0.552^a^
Chorioamnionitis	6 (9.8%)	3 (3.5%)	0.165^b^
Antenatal steroids	47 (77.0%)	60 (70.6%)	0.384^a^
C-section	54 (88.5%)	67 (78.8%)	0.125^a^
Infants’ characteristics			
Gestational age (weeks)	29.2 ± 1.8	29.1 ± 2.0	0.762^c^
Gestational age < 28 weeks	11 (18.0%)	24 (28.2%)	0.154^a^
Male gender	36 (59.0%)	39 (45.9%)	0.117^a^
Positive pressure ventilation at DR	33 (54.1%)	54 (63.5%)	0.252^a^
Advanced resuscitation	Zero	2 (2.4%)	0.510^b^
Birth weight (g)	1,200 ± 324	1,230 ± 357	0.781^c^
Birth weight < 1,000 g	16 (26.2%)	27 (31.8%)	0.469^a^
SNAPPE < 20	35 (57.4%)	49 (57.6%)	0.974^a^
Hypothermia at NICU admission	25 (41.0%)	35 (41.2%)	0.981^a^
Events during the first three DOL			
≥ 1 hypothermia episode	47 (77.0%)	68 (80.0%)	0.667^a^
Mechanical ventilation by tracheal cannula	34 (55.7%)	45 (52.9%)	0.738^a^
Acidosis (pH < 7.2)	7 (11.5%)	18 (21.2%)	0.125^a^
Arterial pCO_2_ > 60 mmHg	4 (6.6%)	10 (11.8%)	0.292^a^
MAP >10 cmH_2_O	8 (13.1%)	4 (4.8%)	0.072^a^
≥ 1 surfactant dose	28 (45.9%)	41 (48.2%)	0.781^a^
Tracheal tube change	2 (3.3%)	7 (8.2%)	0.305^b^
≥ 1 apnea episode	19 (31.1%)	34 (40.0%)	0.273^a^
Systemic hypotension	16 (26.2%)	27 (31.8%)	0.469^a^
PDA pharmacological closing	20 (32.8%)	3 (3.5%)	< 0.001^a^
Hypoglycemia	6 (9.8%)	5 (5.9%)	0.527^b^
Thrombocytopenia < 150,000/mm^3^	18 (29.5%)	26 (30.6%)	0.888^a^
Opioid analgesia	20 (32.8%)	38 (44.7%)	0.147^a^
Adherence to bundle in the first three DOL			
Supine body positioning	18 (29.5%)	71 (83.5%)	< 0.001^a^
Neutral head positioning	16 (26.2%)	69 (81.2%)	< 0.001^a^
Tracheal aspiration	32 (52.5%)	33 (38.8%)	0.102^a^
Respiratory physical therapy maneuvers	12 (19.7%)	zero	< 0.001^a^
CSF collection	31 (50.8%)	9 (10.6%)	< 0.001^a^
Deaths during hospital stay	6 (9.8%)	6 (7.1%)	0.547^a^

^a^chi-square test; ^b^Fisher’s exact test; ^c^t test; DR = delivery room; SNAPPE = score of clinical severity during the first 12 hours of life; moderate hypothermia: axillary temperature < 36 °C; NICU = neonatal intensive care unit; DOL = days of life; MAP = mean airway pressure; systemic hypotension: need for vasopressors or volume expansion; PDA = persistent ductus arteriosus; CSF = cerebrospinal fluid.

The incidence of PIVH of any grade at three days of age was 41% among the infants in the pre-intervention group and 29% in the post-intervention group (P = 0.146). These values were 5% and 3% (P = 0.281), respectively, for grade III/IV lesions on the third day of life. Comparing the incidence of PIVH during the infants’ hospital stay, post-intervention reductions were found both for PIVH of any grade (66% versus 49%; P = 0.05) and for grade III/IV lesions (16% versus 6%; P = 0.03).

The results from the logistic regression analysis performed to investigate risk factors associated with occurrence of PIVH of any grade during the hospital stay are described in Table 2. In model one, being born after bundle implementation reduced the odds of PIVH of any grade during the hospital stay by 71% after adjustment for possible confounding variables. When the pre and post-intervention variable was replaced by variables representing the bundle measures, CSF collection within the first 72 hours increased the odds of PIVH of any grade during the hospital stay fourfold, after adjustment for all variables included in the model.

**Table 2.** Logistic regression analysis to identify factors associated with the dependent variable, i.e. peri/intraventricular hemorrhage of any grade during the hospital stay

	Odds ratio	95% CI	P-value
Model 1^a^			
Post-bundle period	0.289	0.128-0.653	0.003
C-section	0.166	0.053-0.515	0.002
Gestational age < 28 weeks	3.581	1.313-9.765	0.013
Thrombocytopenia < 150,000/mm^3^	3.134	1.246-7.881	0.015
Systemic hypotension	4.664	1.768-12.301	0.002
Model 2^b^			
C-section delivery	0.191	0.061-0.598	0.004
Gestational age < 28 weeks	4.403	1.578-12.283	0.005
Systemic hypotension	4.824	1.820-12.788	0.002
Thrombocytopenia < 150,000/mm^3^	3.487	1.361-8.936	0.009
CSF collection	4.102	1.634-10.298	0.003

95% CI = 95% confidence interval; CSF = cerebrospinal fluid.

^a^Model 1: adjusted for infant gender, cesarean delivery, SNAPPE (score of clinical severity during the first 12 hours of life) < 20, antenatal corticoids, 5-minute Apgar score < 7, birth weight < 1,000 g, ventilation through tracheal cannula during the first three days of life, acidosis episode within the first three days of life, one apnea episode within the first three days of life, pharmacological ductal closing within the first three days of life, and use of opioid analgesia during the first three days of life (Hosmer-Lemeshow: P = 0.341).

^b^Model 2: this model initially included the same variables as Model 1, but afterwards, “bundle period” was removed, and variables relating to adherence to the bundle during the first three days of life (body positioning, head positioning, tracheal aspiration, physical therapy and CSF collection) were introduced (Hosmer-Lemeshow: P = 0.389).

The results from the sensitivity analysis performed to investigate risk factors associated with occurrence of PIVH of any grade at three days of age showed that after adjustment for gestational age, mode of delivery, and systemic hypotension in the first three days of life, being born after bundle implementation reduced the odds of having PIVH of any grade at three days of age by 68% (odds ratio, OR 0.32; 95% confidence interval, CI 0.14-0.75). When the pre and post-intervention variable was replaced by variables representing the bundle measures, maintenance of neutral head position reduced the odds of PIVH of any grade at three days of age by 68% (OR 0.32; 95% CI 0.13-0.77), after adjustment for confounders.

## DISCUSSION

This observational study showed that after adjustment for confounding variables, being born after bundle implementation reduced the odds of PIVH of any grade at three days of age and during the hospital stay by approximately 70%. Among the bundle measures, maintenance of head neutral position stood out as a protective factor against occurrence of PIVH at three days of age, and delaying CSF collection until a more stable period of the neonatal cardiopulmonary transition, i.e. after the first 72 hours of life, was protective against occurrence of PIVH during the hospital stay. The outcome of any PIVH was used in the analysis on associated risk factors because we had few infants with PIVH grades III/IV. By preventing any grade of PIVH, we were also preventing severe PIVH as a consequence. The number of deaths did not increase between the two periods.

The results from the present study corroborate data in the literature indicating that a set of simultaneous measures aimed at improving the quality of neonatal care might have a significant impact with regard to PIVH prevention. In 2003, Carteaux et al.^14^ implemented a set of practices at five institutions to reduce the incidence of intracranial hemorrhage and periventricular leukomalacia in very low birth weight infants. These practices included antenatal betamethasone administration; optimization of peripartum management; optimization of direct clinical management by neonatologists and trained nurses; implementation of measures to minimize pain and stress responses; maintaining neutral head position in the first days of life; judicious treatment of hypotension by means of fluid volume therapy followed by use of inotropic agents as needed; judicious use of indomethacin to help close cases of PDA; optimization of respiratory management; limited use of sodium bicarbonate; and judicious postnatal use of dexamethasone. While difficulties in adhering to these suggested practices have been reported,^22^ their impact on the incidence of PIVH has not yet been elucidated.

In 2013, Schmid et al.^8^ showed that a program of preventive measures within perinatal care reduced the incidence of PIVH from 22% to 10% among very low birth weight preterm infants. De Bijl-Marcus et al., in 2020, published the results from a bundle of nursing interventions that consisted of posture interventions and avoidance of rapid intravenous/arterial flushes and rapid arterial blood withdrawal.^15^ Use of the bundle was associated with lower risk of developing any degree of PIVH, cystic periventricular leukomalacia and/or mortality (adjusted OR 0.42; 95% CI 0.27 to 0.65). ^15^ Other studies, such as the one conducted by Lee et al.,^13^ sought to improve the quality of care delivered and reduce occurrences of PIVH grades III/IV and/or leukomalacia as a secondary outcome among premature infants with gestational ages less than 29 weeks. The practices implemented to reduce severe neurological injury consisted of delayed cord clamping, antenatal use of magnesium sulfate, minimal use of volume expanders and minimal use of inotropes. However, the results did not show any significant reduction in severe neurological injury.

This study, as well as the study conducted by Schmid et al.,^8^ focused predominantly on measures aimed at stabilizing infants during the period of cardiopulmonary transition, when infants are vulnerable to hemodynamic fluctuations that impact the germinal matrix.^23^ Despite the positive result, it should be highlighted that the incidence of PIVH at the NICU studied here was much higher than the incidence of 20-25% reported in the United States for infants with birth weight < 1,500 g.^24^ Measures aimed at improving the quality of hospital care may have greater impact on conditions of higher prevalence.

In designing this study, it was decided to exclude infants who died within the first 168 hours of life because death and PIVH are competing risks. The best strategy for analyzing competing risks is to use composite outcomes, but these are difficult to use and lead to errors of interpretation and sample size calculation.^25^ Moreover, composite outcomes are generally inadequate, thus implying that the results apply to the individual components of the composite outcome rather than only to the overall composite.^26^ Since nearly all occurrences of PIVH develop within the first week after birth, it was therefore decided, in this study, to exclude infants who died before the occurrence of PIVH. We also excluded infants who underwent surgical procedure during this same period, because the procedure itself and the anesthetic procedure might have biased our outcome.

With regard to the mechanisms underlying PIVH, turning the infant’s head to one side might occlude or obstruct drainage of the ipsilateral jugular vein, which might consequently increase local venous congestion.^17^ The results from the present study stress the relevance of this pathophysiological mechanism, given that after adjustment for possible confounding factors, the protection afforded by neutral head position during the first 72 hours of life was significant. Romantsik et al. conducted a systematic review to assess whether head midline position would be more effective than any other position for preventing or extending PIVH and they did not find any significant differences in the outcomes.^27^ They included a total of 110 infants in their review, from two randomized controlled trials, and found that the difference in the risk of PIVH, comparing the supine midline head position with the supine rotated head position, was 0.03 (95% CI: -0.13 to 0.18).^27^ The results from this systematic review did not provide a definitive answer to the review question.^27^ Another review of the literature was conducted by Malusky and Donze^17^ to evaluate changes in cerebral hemodynamics in response to position changes. They found evidence to recommend a midline head position during the first 72 hours of life among infants with gestational ages of less than 32 weeks.^17^ Some studies have indicated that it is important to maintain an elevated position, in addition to the midline neutral position.^28^ The protocol at the NICU of the present study is to admit and maintain all preterm infants in a position of head elevation (approximately 15°).

CSF collection requires considerable postural manipulation, including the position of the infant’s head, which might result in fluctuations in cerebral blood flow. Some studies have shown that during CSF collection in newborn infants, the arterial blood partial pressure of oxygen decreases and that of carbon dioxide increases.^20^^,^^29^ These factors, together with the pain caused by the procedure,^30^ might alter cerebral blood flow. A retrospective study that included 106,461 very low birth weight infants found an association between lumbar puncture for CSF collection in the first three days of life and occurrence of PIVH grades III/IV.^31^ At birth, the risk of PIVH grades III/IV was more than double for infants who received the procedure (adjusted OR 2.64, 95% confidence interval 1.96-3.54).^31^

The results from the present study suggest that care practices that can minimize fluctuations in cerebral blood flow among very low birth weight preterm infants in the first days of life might result in significant reductions in intracranial hemorrhage during that period and throughout the hospital stay. Despite this finding, postponing the diagnosing of early meningitis may be an undesirable effect of postponing lumbar puncture. One third or more of infants with meningitis have a negative blood culture.^32^^,^^33^ However, the incidence of early onset meningitis is much lower than the incidence of late onset meningitis (1.1/1000 versus 13.9/1000 in very low birth weight infants in 2005 and 2001, respectively).^32^^,^^33^^,^^34^ The consequences of postponing CSF collection in infants with suspected early onset sepsis has not been studied. Therefore, the risk-benefit relationship of early CSF collection needs to be individually balanced.

The present study had several limitations, and the main one among these was its methodological design. Because it was a cohort study involving two periods of time, the changes introduced in neonatal care between the periods may have been confounding factors. In this regard, the main change in the NICU between the two periods was the introduction of functional echocardiography, which became routine for assessing the presence and repercussions of PDA. As a result, the number of infants requiring pharmacological or surgical treatment for PDA was significantly reduced, as their treatment became more conservative, which could have influenced the incidence of PIVH. For this reason, the statistical analysis took this change into consideration, and the analysis was also adjusted for prenatal, delivery and postnatal factors that would affect the pathogenesis of PIVH. Although the two cohorts represent clinical practices that are now more than five years old, these practices are still up-to-date and the comparison between these two periods can help clinicians to better understand the impact of these practices. Another limitation was that the diagnosis of PIVH was not made by a single US technician. Nevertheless, as the study was conducted in a teaching hospital and, thus, the final US reports were systematically reviewed by the professor in charge of teaching the technique for head US, the diagnosis can be considered to have been uniform throughout the study.

## CONCLUSIONS

These limitations notwithstanding, the present study demonstrated that a set of measures to prevent PIVH in preterm infants with gestational age less than 32 weeks was effective in decreasing the incidence of all grades of intraventricular hemorrhage. Implementation of these measures mainly requires education for the NICU staff regarding the need for care practices that minimize fluctuations in cerebral blood flow. These measures are low-cost and easy to implement, although they are difficult to maintain in the long run because consistent implementation requires staff to be continuously reminded of their value.
